# Detection of serum MMP-7 and MMP-9 in cholangiocarcinoma patients: evaluation of diagnostic accuracy

**DOI:** 10.1186/1471-230X-9-30

**Published:** 2009-04-30

**Authors:** Kawin Leelawat, Sompong Sakchinabut, Siriluck Narong, Jerasak Wannaprasert

**Affiliations:** 1 Department of Surgery, Rajavithi Hospital, Bangkok 10400, Thailand; 2 Department of Surgery, College of Medicine, Rangsit University, Bangkok 10400, Thailand

## Abstract

**Background:**

Cholangiocarcinoma is an aggressive tumor with a tendency for local invasion and distant metastases. Timely diagnosis is very important because surgical resection (R0) remains the only hope for a cure. However, at present, there is no available tumor marker that can differentiate cholangiocarcinoma from benign bile duct disease. Previous studies have demonstrated that matrix metalloproteinase (MMP)-7 and MMP-9 are frequently expressed in cholangiocarcinoma specimens.

**Methods:**

This study was designed to determine whether the serum levels of MMP-7 and MMP-9 can discriminate cholangiocarcinoma patients from benign biliary tract disease patients in comparison to carcinoembryonic antigen (CEA) and carbohydrate antigen 19-9 (CA19-9). We measured the level of CEA, CA19-9, MMP-7 and MMP-9 in the serum of 44 cholangiocarcinoma and 36 benign biliary tract diseases patients.

**Results:**

Among the serum levels of CEA, CA19-9, MMP-7 and MMP-9, only the serum MMP-7 level was significantly higher in the patients with cholangiocarcinoma (8.9 ± 3.43 ng/ml) compared to benign biliary tract disease patients (5.9 ± 3.03 ng/ml) (*p *< 0.001). An receiver operating characteristic (ROC) curve analysis revealed that the detection of the serum MMP-7 level is reasonably accurate in differentiating cholangiocarcinoma from benign biliary tract disease patients (area under curve = 0.73; 95% CI = 0.614–0.848). While the areas under the curve of the ROC curves for CEA, CA19-9 and MMP-9 were 0.63 (95% CI = 0.501–0.760), 0.63 (95% CI = 0.491–0.761) and 0.59 (95% CI = 0.455–0.722), respectively.

**Conclusion:**

Serum MMP-7 appears to be a valuable diagnostic marker in the discrimination of cholangiocarcinoma from benign biliary tract disease. Further prospective studies for serum MMP-7 measurement should be carried out to further investigate the potential of this molecule as a biomarker of cholangiocarcinoma.

## Background

The incidence of and mortality rate for cholangiocarcinoma varies considerably among different geographic regions, with the highest incidence being observed in Southeast Asia, especially in Thailand [1]. In the United States, the most commonly recognized risk factor for cholangiocarcinoma is primary sclerosing cholangitis (PSC) [2,3]. However, in Southeast Asia and especially in Thailand, infection with hepatobiliary flukes (*Opisthorchis viverrini*) is the most common risk factor for cholangiocarcinoma [4]. Therapeutic options for cholangiocarcinoma have been limited since this type of cancer responds poorly to chemotherapy and radiation therapy. Surgery is perhaps the only effective treatment for cholangiocarcinoma. Five-year survival, which typically has a rate between 32% and 50%, is achieved by only a small number of patients when negative histological margins are attained at the time of surgery [5]. To improve the survival rate, patients must be diagnosed and treated as early in the disease onset as possible.

To properly diagnose cholangiocarcinoma, it is very difficult to get to the tissue due to the tumor location and the desmoplastic reaction. In addition, this tumor typically grows along the bile duct without expanding from the bile ducts as a forming mass. Computed tomography (CT), ultrasound, and magnetic resonance imaging (MRI) often miss this lesion [6]. Therefore, identification of tumor markers in the serum would be beneficial in the clinical management of this disease. To date, there are two common tumor markers used for detecting cholangiocarcinoma, carcinoembryonic antigen (CEA) and carbohydrate antigen 19-9 (CA19-9). CEA is unspecific and can be elevated in the setting of other gastrointestinal or gynecologic malignancies or other bile duct pathologies, such as cholangitis and hepatolithiasis [7]. Previous studies have demonstrated that the sensitivity and specificity of a CA 19-9 value >100 U/ml for cholangiocarcinoma in primary sclerosing cholangitis (PSC) are 89% and 86%, respectively [8,9]. However, a cut-off of the CA 19-9 value at 100 U/ml resulted in a sensitivity of only 53.0–67.5% for diagnosing cholangiocarcinoma in patients without PSC [10,11]. In addition, a previous study demonstrated that the level of serum CA19-9 is dependent on the severity of the bile duct obstruction and the degree of cholangitis. An increase in the serum level of CA19-9 can be detected even in benign bile duct diseases [12,13]. Therefore, novel tumor markers should be investigated to better diagnose cholangiocarcinoma in patients with or without PSC.

Typically, tumor cells invade the basement membrane by secreting enzymes that digest the extracellular matrix proteins. These enzymes are known as matrix metalloproteinase (MMPs). MMPs are zinc-dependent endopeptidases. They are involved in the turnover and degradation of the extracellular matrix (ECM) components and basement membranes [14]. Recently, Itatsu K, et al. examined the expression of MMPs in surgically resected specimens of cholangiocarcinoma using an immunohistochemical method and found that 47.5 and 75.8% of these specimens expressed MMP-9 and MMP-7, respectively [15]. Previous studies have demonstrated that MMP-9 can be detected in the serum of gastric cancer patients and MMP-7 is increased in colorectal, ovarian and renal cancer patients [16-19]. Therefore, detection of MMP-9 and MMP-7 in the blood circulation may be useful for the clinical diagnosis of cholangiocarcinoma. To date, there is no published study on the detection of serum levels of MMP-9 and MMP-7 in cholangiocarcinoma patients. The objective of this study was to determine the accuracy of detecting serum levels of MMP-9 and MMP-7 for the diagnosis of cholangiocarcinoma in patients without primary sclerosing cholangitis.

## Methods

### Patients and samples

Pre-treatment fasting serum samples (n = 80) were collected from obstructive jaundice patients who underwent endoscopic retrograde cholangiography (ERCP) or biliary tract surgery at Rajavithi Hospital. All patient sera and clinical information were obtained with patient consent after approval by Rajavithi Ethics Committee. Thirty-six patients were diagnosed with benign biliary tract diseases, and 44 patients were diagnosed as having cholangiocarcinoma by one of the following criteria: 1) tissue biopsy (n = 7), 2) cytology (n = 17), and 3) radiological finding (helical CT scan or MRI) and clinical observation to identify the progression of the tumor at follow up (n = 20). Serum samples from these patients were separated by centrifugation within 2 h and frozen at -80°C. The biochemical studies of serum samples, including AST, ALT, total and direct bilirubin, alkaline phosphatase (ALP), CEA and CA19-9, were measured using routine automated methods in the Pathological Laboratory at Rajavithi Hospital.

### Measurement of serum MMP-7 and MMP-9

Serum MMP-9 and MMP-7 levels were measured using an enzyme-linked immunosorbent assay (ELISA) kit (R&D Systems, Minneapolis, MN). The diluted serum samples were added in duplicate to 96-well plates coated with the MMP-9 or MMP-7 antibody and incubated at room temperature for 2 h. After washing three times with washing buffer, the conjugated secondary antibody was added, and the plate was further incubated for 2 h. Plates were washed again prior to incubation with the substrate solution for 1 h. The amplifier solution was then added, and the plate was incubated for an additional 30 min. All incubation cycles were performed at room temperature. Following termination of the reaction with the stop solution (1 N sulfuric acid), the optical density was measured at 490 nm using a spectrophotometric microplate reader. The concentration of MMP-9 and MMP-7 in each sample was calculated from a standard curve.

### Statistical analysis

Comparison between the quantitative variables was performed by using *Mann-Whitney U *or Student's t-test, as appropriate. Qualitative variables were reported as counts, and comparisons between independent groups were performed by using by Pearson Chi-square. The diagnostic accuracy of each of the candidate biomarkers was evaluated using receiver operating characteristic (ROC) curve analysis, which correlates true- and false-positive rates [sensitivity and (1-specificity)]. In addition, an area under the ROC curve (AUC) with 95% confidence intervals (CI) was calculated for each marker. The optimal cut-off points for MMP-9 and MMP-7 were selected based on the ROC curve analysis. Sensitivity, specificity, positive predictive value and negative predictive value were calculated using a 2 × 2 table of the collected data.

## Results

### Patient Characteristics

In cholangiocarcinoma cases, there were 12 cases of intrahepatic cholangiocarcinoma and 32 cases of perihilar cholangiocarcinoma. Primary or secondary common bile duct stones (*78%; n *= 28) were the most common diseases in the control patients. The clinical characteristics of the patients in this study are shown in Table 1. No statistically significant differences were found among the data of the patients considered as controls and those with cholangiocarcinoma regarding gender, age, serum albumin, globulin and ALT levels. However, the level of serum AST, bilirubin and alkaline phosphatase were significantly higher in cholangiocarcinoma patients than in controls (*Mann-Whitney U test; p *< 0.05).

**Table 1:** Clinical characteristics of the patients with benign biliary tract disease (control) and cholangiocarcinoma

	Control (n = 36)	Cholangiocarcinoma (n = 44)	*p *value
Age (Yr)	54 ± 14.5	59 ± 12.9	0.130
Sex (Male:Female)	15:16	26:18	0.248^#^
Total bilirubin (mg/dL)	4.2 ± 5.53	14.6 ± 11.34	<0.001*
Direct bilirubin (mg/dL)	2.6 ± 3.75	10.3 ± 8.47	<0.001*
Albumin (g/dL)	3.8 ± 0.61	3.1 ± 0.68	0.050
Globulin (g/dL)	3.6 ± 0.73	4.1 ± 0.93	0.253
AST (U/L)	65.4 ± 53.80	183.9 ± 378.82	0.012*
ALT (U/L)	75.0 ± 77.72	101.4 ± 14.49	0.615
ALP (IU/L)	318.6 ± 349.65	551.8 ± 526.04	0.001*

Quantitative variables are presented as the means ± standard deviation. #; Pearson Chi-square was used to compare between two groups, *; the level of serum total bilirubin, direct bilirubin, AST and ALP were significantly higher in cholangiocarcinoma patients than in controls (*Mann-Whitney U test; p *< 0.05).

### Detection of CEA and CA19-9 in serum of cholangiocarcinoma and benign obstructive jaundice patients

**Figure 1:**
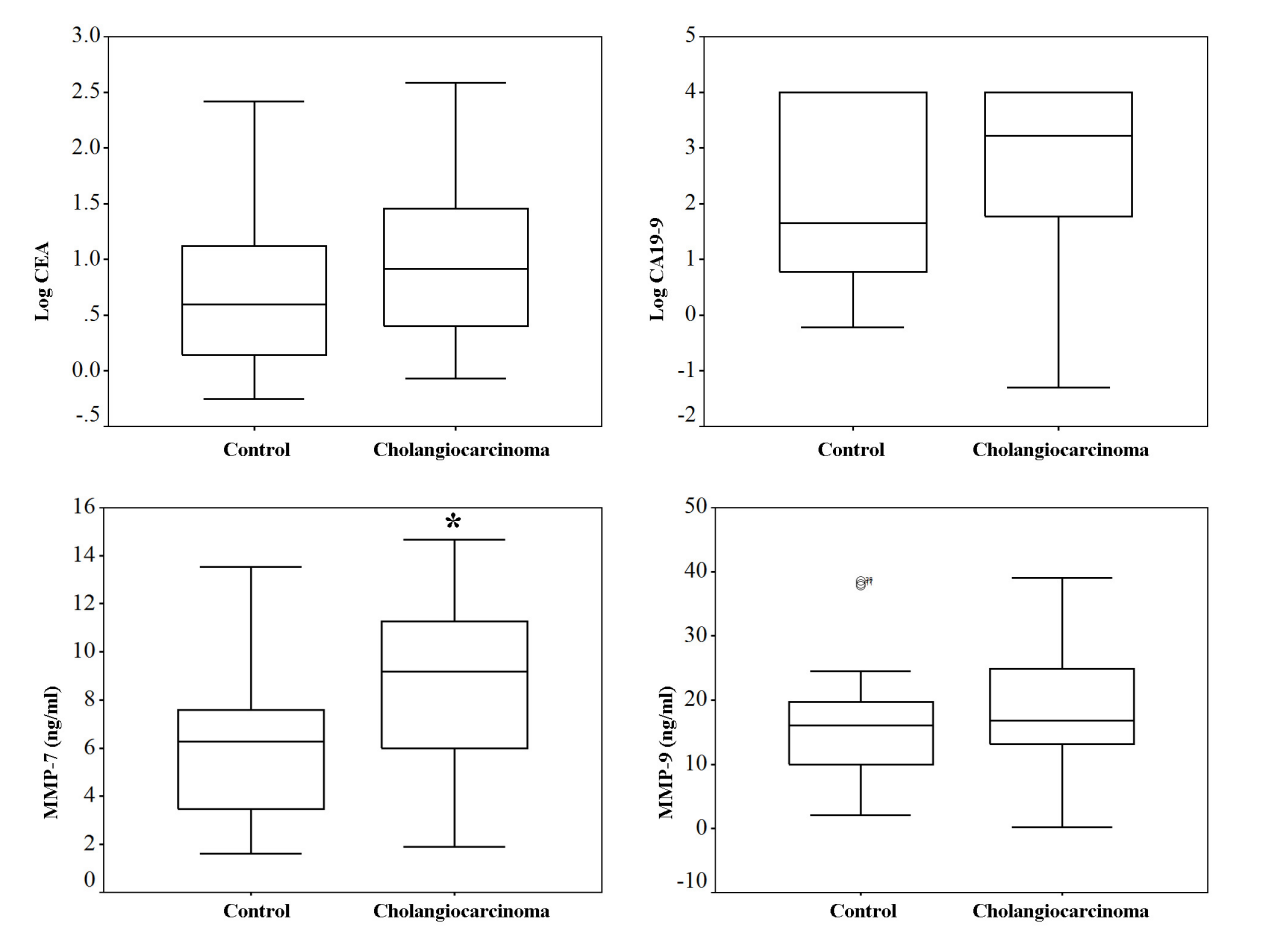
**Serum levels of CEA, CA19-9, MMP-7 and MMP-9 in cholangiocarcinoma and control (benign biliary tract disease) patients**. Box plots comparing levels of CEA, CA19-9, MMP-7 and MMP-9 are demonstrated. Levels of MMP-7 and MMP-9 are presented as ng/ml, while CEA and CA19-9 are presented with the log data to accommodate the wide range. *; Only the value for MMP-7 between the two groups is significantly different (Student's t-test; *p *< 0.001).

The median CEA and CA19-9 values in the control group were 3.96 ng/ml (range; 0.56–260.24) and 45.88 U/ml (range 0.60–10000.00), respectively. The median CEA and CA19-9 values in the cholangiocarcinoma group were 8.27 ng/ml (range; 0.85–131.70) and 2176.00 U/ml (range; 0.50–10000.00), respectively. However, there was no statistically significant difference in the levels of these two markers between the control and cholangiocarcinoma patients (*Mann-Whitney U test; p *= 0.057 for CEA and *p *= 0.056 for CA19-9). These data are shown in Figure 1. We used a CEA cut-off value of 5 ng/ml and a CA19-9 cut-off value of 100 U/ml because these have been the suggested cut-off value for the diagnosis of cholangiocarcinoma [7]. Using a CEA cut-off value of 5 ng/ml, the sensitivity was determined to be 58.54% (CI 95% 43.37 – 72.24), and the specificity was determined to be 62.50% (CI 95% 45.25 – 77.07). Using a CA19-9 cut-off value of 100 U/ml, the sensitivity was determined to be 70.45% (CI 95% 55.78 – 81.84), and the specificity was determined to be 63.64% (CI 95% 46.62 – 77.81).

### Detection of MMP-9 and MMP-7 in serum of cholangiocarcinoma and benign obstructive jaundice patients

There was no statistically significant difference in the levels of MMP-9 between the control (mean ± SD; 16.5 ± 9.30 ng/ml) and cholangiocarcinoma patients (mean ± SD; 18.9 ± 8.55 ng/ml), (Student's t-test; *p *= 0.251, 95% CI -1.74–6.55). In contrast, the serum MMP-7 values in the cholangiocarcinoma patients (mean ± SD; 8.9 ± 3.43 ng/ml) were significantly higher than those in the control patients (mean ± SD; 5.9 ± 3.03 ng/ml), (Student's t-test; *p *< 0.001, 95% CI 1.34–4.47).

### ROC curve analysis for CEA, CA19-9, MMP-9 and MMP-7 for diagnosis of cholangiocarcinoma

**Figure 2:**
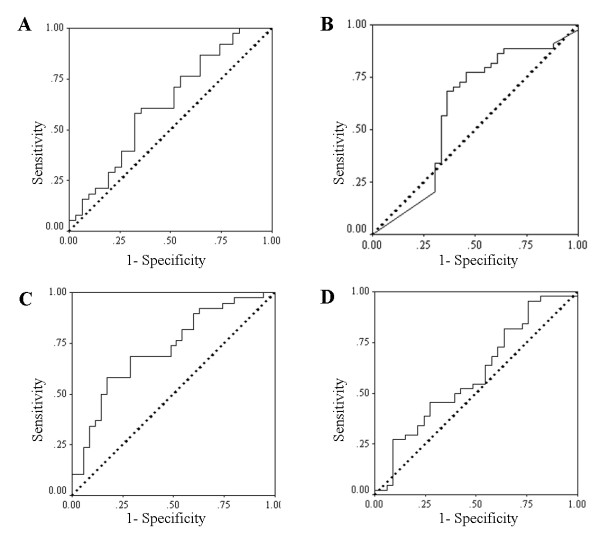
**ROC curve analyses of CEA, CA19-9, MMP-9 and MMP-7 for the diagnosis of cholangiocarcinoma**. The diagnostic accuracy of each biomarker, in terms of its sensitivity and specificity, are presented by receiver operating characteristic (ROC) curve analysis. Figures 2A, 2B, 2C and 2D correspond to **CEA, CA19-9, MMP-7 and MMP-9**. Only the area under the curve (AUC) of the ROC for MMP-7 is significantly higher than a chance value (0.5).

An ROC curve analysis (Figure 2) was used to calculate an area under the curve (AUC) of 0.63 (CI 95% 0.501 – 0.760) and of 0.63 (CI 95% 0.491 – 0.761) for CEA and CA19-9, respectively. Additionally, an ROC curve analysis was used to calculate an area under the curve of 0.59 (CI 95% 0.455 – 0.722) and of 0.73 (CI 95% 0.614 – 0.848) for MMP-9 and MMP-7, respectively. When comparing the AUC of the ROC curve for CEA, CA19-9, MMP-9 and MMP-7 with a chance value equal to 0.5 (the worst value of AUC of ROC), only the AUC of the ROC for MMP-7 is significantly higher than 0.5 (*p *= 0.001). The sensitivity and specificity for CEA, CA19-9, MMP-9 and MMP-7 are presented in Table 2.

**Table 2:** Performance of the biomarkers for the diagnosis of cholangiocarcinoma

Biomarker (cut-off value)	Sensitivity (%) (95% CI)	Specificity (%) (95% CI)	PLR (95% CI)	NLR (95% CI)
**CEA** **(3 ng/ml)**	70.73 (55.52–82.39)	43.75 (28.17–60.67)	1.26 (0.87–1.81)	0.67 (0.36–1.24)
**CEA** **(5 ng/ml)**	58.54 (43.37–72.24)	62.50 (45.25–77.07)	1.56 (0.93–2.62)	0.66 (0.42–1.04)
**CA19-9** **(35 U/ml)**	81.82 (68.04–90.49)	48.48 (32.50–64.78)	1.59 (1.11–2.27)	0.38 (0.18–0.77)
**CA19-9** **(100 U/ml)**	70.45 (55.78 – 81.84)	63.64 (46.62–77.81)	1.94 (1.19–3.16)	0.46 (0.28–0.78)
**MMP-9** **(15.0 ng/ml)**	63.64 (48.87–76.22)	41.94 (26.42–59.23)	1.67 (0.93–3.01)	0.59 (0.35–0.98)
**MMP-9** **(20.0 ng/ml)**	34.10 (21.88–48.86)	74.19 (56.75–86.30)	1.32 (0.64–2.73)	0.89 (0.66–1.20)
**MMP-7** **(6.0 ng/ml)**	76.32 (60.79–87.01)	46.88 (30.87–63.55)	1.44 (0.99–2.08)	0.51 (0.26–1.00)
**MMP-7** **(7.4 ng/ml)**	63.16 (47.28–76.62)	71.88 (54.63–84.44)	2.25 (1.23–4.11)	0.51 (0.32–0.82)

The sensitivity, specificity, positive and negative likelihood ratio (LR) as well as their 95% confidence interval (CI) for each marker is presented. The likelihood ratio is the ratio of true and false positives (sensitivity and 1-specificity respectively), where the higher values reflect the probability of a better performance. (PLR, positive likelihood ratio; NLR, negative likelihood ratio; 95% CI, 95% confidence interval)

Due to the significant difference of the serum AST, ALP, total bilirubin and direct bilirubin between the control and cholangiocarcinoma patients, we investigated the correlation between the values of these blood chemistries and the values for CEA, CA19-9, MMP-9 and MMP-7. The results showed that none of these parameters was significantly correlated (*p *> 0.05) (see Addition file 1 and Addition file 2). To determine whether the values of serum MMP-9 and MMP-7 were predictive of cholangiocarcinoma independently of other tumor markers, we carried out a logistic regression analysis. In a multivariable model using MMP-9 (cut-off value = 15 ng/ml), MMP-7 (cut-off value = 7.4 ng/ml), CEA (cut-off value = 5 ng/ml), CA19-9 (cut-off value = 100 U/ml), MMP-9 (an adjusted odds ratio = 3.76; 95% CI = 1.05–13.47; *p *= 0.04), MMP-7 (an adjusted odds ratio = 5.33; 95% CI = 1.55–18.31; *p *= 0.008) and CA19-9 (an adjusted odds ratio = 4.60; 95% CI = 1.23–17.30; *p *= 0.02) were the independent predictors of cholangiocarcinoma, whereas CEA was not.

## Discussion

The need for better tests to diagnose and screen for patients with cholangiocarcinoma is an important issue that must be addressed to improve the treatment results for these patients. Unfortunately, no specific serum tumor markers have been identified for this disease.

Based on the results of our study, the sensitivity and specificity of CEA as a marker for detecting cholangiocarcinoma are 58.54% and 62.50%, respectively. This is consistent with previously published studies that reported that the sensitivity and specificity of CEA for detecting cholangiocarcinoma were 33–84% and 33–100%, respectively [7,20]. Previous articles have addressed the accuracy of CA19-9 in the identification of cholangiocarcinoma. A previous study identified cholangiocarcinoma with a sensitivity of 67.5% and a specificity of 86.8% when a cut-off value of 100 U/ml for CA19-9 was used and a sensitivity of 77.9% and a specificity of 76.3% when a cut-off value of 35 U/ml for CA19-9 was used [10]. In our series, we found that the sensitivity was 70.45% and the specificity was 63.64% when using a cut-off value of 100 U/ml for CA19-9. However, the AUC of the ROC curve for CA19-9 was only 0.63 in the discrimination of cholangiocarcinoma in our study. Therefore, when the cut-off value was changed to 35 U/ml, the specificity markedly decreased (81.82% of sensitivity and 48.48% of specificity). We suggest that the differences among the patients should be concerned. In the study published by John, A. R., et al, 25 patients with benign liver tumors and 13 patients with benign bile duct strictures were used as a control group [10]. However, in our studies, all the subjects in the control group had been diagnosed with benign bile duct diseases. The reason that we used patients with benign bile duct diseases as a control group was because the symptoms of cholangiocarcinoma are similar to the symptoms of benign bile duct diseases in our clinical setting.

We observed that most of the cholangiocarcinoma patients were suffering from the invasiveness of the cholangiocarcinoma cells into the adjacent organs. The mechanism by which cancer cells invade the surrounding tissue requires the breakdown of the extracellular matrix and the subsequent migration of the cancerous cells through the degraded structures [14]. Because extracellular matrix remodeling is the major activity of a family of enzymes known as matrix metalloproteinases (MMPs), these enzymes were investigated for their contributions to the malignant phenotype in cholangiocarcinoma patients. Previous studies have demonstrated that the expression of MMP-9 and MMP-7 can be detected in cholangiocarcinoma specimens [21-23]. Therefore, in our study, the accuracy of serum MMP-9 and MMP-7 levels were investigated in an effort to find a reliable serum marker that can discriminate the benign biliary tract diseases from cholangiocarcinoma.

There are numerous studies that demonstrate that the serum level of MMP-9 is significantly elevated in many types of cancers, including breast cancer, esophageal cancer, and lung cancer [24-26], but previous reports have shown that the incidence of MMP-9 expression in cholangiocarcinoma specimens is only 9–47.5% [15,22]. Our study demonstrated that there is no statistically significant difference in the serum MMP-9 levels between cholangiocarcinoma patients and control patients. Previous studies revealed that detection of MMP-9 in serum is an artifact representing the release of MMP-9 from leukocytes during the clotting process in the blood collection tube [27,28]. The role of circulating MMP-9 in diagnosing cholangiocarcinoma should be further investigated by collecting the plasma instead of serum and the assay should be performed without long delay [29].

Previous studies have demonstrated that cholangiocarcinoma specimens frequently express MMP-7 (75.8–100%) [15,21]. As far as we are aware, no other published investigation is available that uses the serum MMP-7 level to diagnose cholangiocarcinoma. Our study shows that the serum MMP-7 level is significantly higher in patients with cholangiocarcinoma than with benign biliary tract diseases. MMP-7 is the smallest of the MMPs and has been demonstrated to degrade or process a variety of matrix and nonmatrix molecules. Unlike most MMPs, which are expressed by stromal cells, MMP-7 is principally expressed by epithelial cells [30]. A previous study reported that the serum MMP-7 level was significantly elevated in patients with ovarian cancer and advanced colorectal cancer [16,31]. We suggest that MMP-7 might be detected in many cancers that originate from epithelial cells. In addition, we also found that the accuracy of the serum MMP-7 level for the diagnosis of cholangiocarcinoma is better than the serum level of MMP-9, CEA and CA19-9, as observed by calculating the AUC of the ROC curve. Only the AUC of the ROC curve for the serum MMP-7 level is significantly higher than a chance value (0.5). Our study demonstrated that use of serum MMP-7 could identify cholangiocarcinoma patients from benign biliary tract disease patients. However, further larger prospective studies that evaluated the benefit of serum MMP-7 in helping the physician to take decisions on diagnosis cholangiocarcinoma are necessary before the implementation of using serum MMP-7 as a marker for cholangiocarcinoma. Previous studies determined that expression of MMP-7 in cholangiocarcinoma is an unfavorable postoperative prognostic factor for cholangiocarcinoma patients [15]. However, in this study, most of the cholangiocarcinoma patients had been diagnosed with unresectable tumors; only five patients underwent curative resection (R0). Therefore, an analysis for a prognostic factor of cholangiocarcinoma could not be clarified. Further studies that include many cases of resectable cholangiocarcinoma need to be completed before the serum MMP-7 level can be used as a prognostic factor for cholangiocarcinoma.

## Conclusion

The elevation of serum MMP-7 levels could be a very useful tool for the detection of cholangiocarcinoma, especially in those patients with obstructive jaundice.

## Competing interests

The authors declare that they have no competing interests.

## Authors' contributions

KL conceived of, designed, and coordinated the study and the statistical analysis and drafted the manuscript. SS coordinated the study and helped with the statistical analysis. SN carried out the MMP-7 and MMP-9 assays and helped with the statistical analysis and JW coordinated the study.

## Pre-publication history

The pre-publication history for this paper can be accessed here:

http://www.biomedcentral.com/1471-230X/9/30/prepub

## Supplementary Material

Additional file 1 **The correlation between the blood chemistry values and MMP-9 or MMP-7**. This table demonstrates the correlation between the blood chemistry values (total bilirubin, AST, ALP, Log CEA and Log CA19-9) and MMP-9 or MMP-7 in the control and cholangiocarcinoma patients. No significant correlation is identified (*p *> 0.05). Click here for file

Additional file 2 **Scatter plot of the correlation between the blood chemistry values and MMP-9 or MMP-7**. A scatter plot was used to identify the correlation between the blood chemistry values (total bilirubin, AST, ALP, Log CEA and Log CA19-9) and MMP-9 or MMP-7 in the control and cholangiocarcinoma patients. This figure demonstrates that there is no significant correlation (*p *> 0.05) between the blood chemistry values and MMP-9 or MMP-7 in both groups. Click here for file
