# Supplementary material for: Detection of serum MMP-7 and MMP-9 in cholangiocarcinoma patients: evaluation of diagnostic accuracy
Source: BMC Gastroenterol. 2009 Apr 30;9:30. doi: 10.1186/1471-230X-9-30 (PMC2680894; doi:10.1186/1471-230X-9-30)
Supplement: Additional file 1 — The correlation between the blood chemistry values and MMP-9 or MMP-7. This table demonstrates the correlation between the blood chemistry values (total bilirubin, AST, ALP, Log CEA and Log CA19-9) and MMP-9 or MMP-7 in the control and cholangiocarcinoma patients. No significant correlation is identified (p > 0.05). [file 1471-230X-9-30-S1.pdf]

Addition file 1 ; Correlation between blood chemistries and biomarkers

Significant *p*<0.05

			**Total Bilirubin**	**Albumin**	**Globulin**	**AST**	**ALT**	**ALP**	**LOG CA19-9**	**LOG CEA**	**MMP-9**	**MMP-7**
**Control**												
	**LOG CA19-9**	Pearson Correlation	-0.18	0.03	-0.33	-0.13	-0.21	-0.42	1.00	0.30	-0.07	-0.25
		Sig. (2-tailed)	0.34	0.89	0.07	0.49	0.28	0.02	.	0.14	0.70	0.22
	**LOG CEA**	Pearson Correlation	0.11	-0.54	-0.11	0.02	-0.04	0.34	0.30	1.00	-0.02	0.27
		Sig. (2-tailed)	0.61	0.01	0.62	0.92	0.86	0.10	0.14	.	0.93	0.24
	**MMP-9**	Pearson Correlation	-0.08	0.10	0.11	-0.10	0.03	0.00	-0.07	-0.02	1.00	-0.23
		Sig. (2-tailed)	0.66	0.61	0.57	0.62	0.88	0.99	0.70	0.93	.	0.26
	**MMP-7**	Pearson Correlation	0.10	-0.34	0.06	0.27	0.26	0.53	-0.25	0.27	-0.23	1.00
		Sig. (2-tailed)	0.64	0.10	0.77	0.19	0.22	0.01	0.22	0.24	0.26	.
**Cholangiocarcinoma**												
			**Total Bilirubin**	**Albumin**	**Globulin**	**AST**	**ALT**	**ALP**	**LOG CA19-9**	**LOG CEA**	**MMP-9**	**MMP-7**
	**LOG CA19-9**	Pearson Correlation	0.03	-0.14	0.20	-0.01	-0.09	-0.02	1.00	0.14	-0.11	0.31
		Sig. (2-tailed)	0.86	0.36	0.20	0.94	0.57	0.90	.	0.44	0.46	0.07
	**LOG CEA**	Pearson Correlation	0.17	0.04	-0.05	-0.35	0.02	-0.04	0.14	1.00	-0.08	-0.07
		Sig. (2-tailed)	0.34	0.82	0.79	0.05	0.90	0.81	0.44	.	0.66	0.75
	**MMP-9**	Pearson Correlation	-0.19	0.00	0.25	-0.10	-0.09	0.10	-0.11	-0.08	1.00	-0.17
		Sig. (2-tailed)	0.23	0.99	0.11	0.51	0.57	0.51	0.46	0.66	.	0.33
	**MMP-7**	Pearson Correlation	0.20	-0.04	0.15	0.24	-0.04	0.02	0.31	-0.07	-0.17	1.00
		Sig. (2-tailed)	0.28	0.84	0.42	0.19	0.82	0.91	0.07	0.75	0.33	.
