# Supplementary figures and images for: Detection of serum MMP-7 and MMP-9 in cholangiocarcinoma patients: evaluation of diagnostic accuracy
Source: BMC Gastroenterol. 2009 Apr 30;9:30. doi: 10.1186/1471-230X-9-30 (PMC2680894; doi:10.1186/1471-230X-9-30)

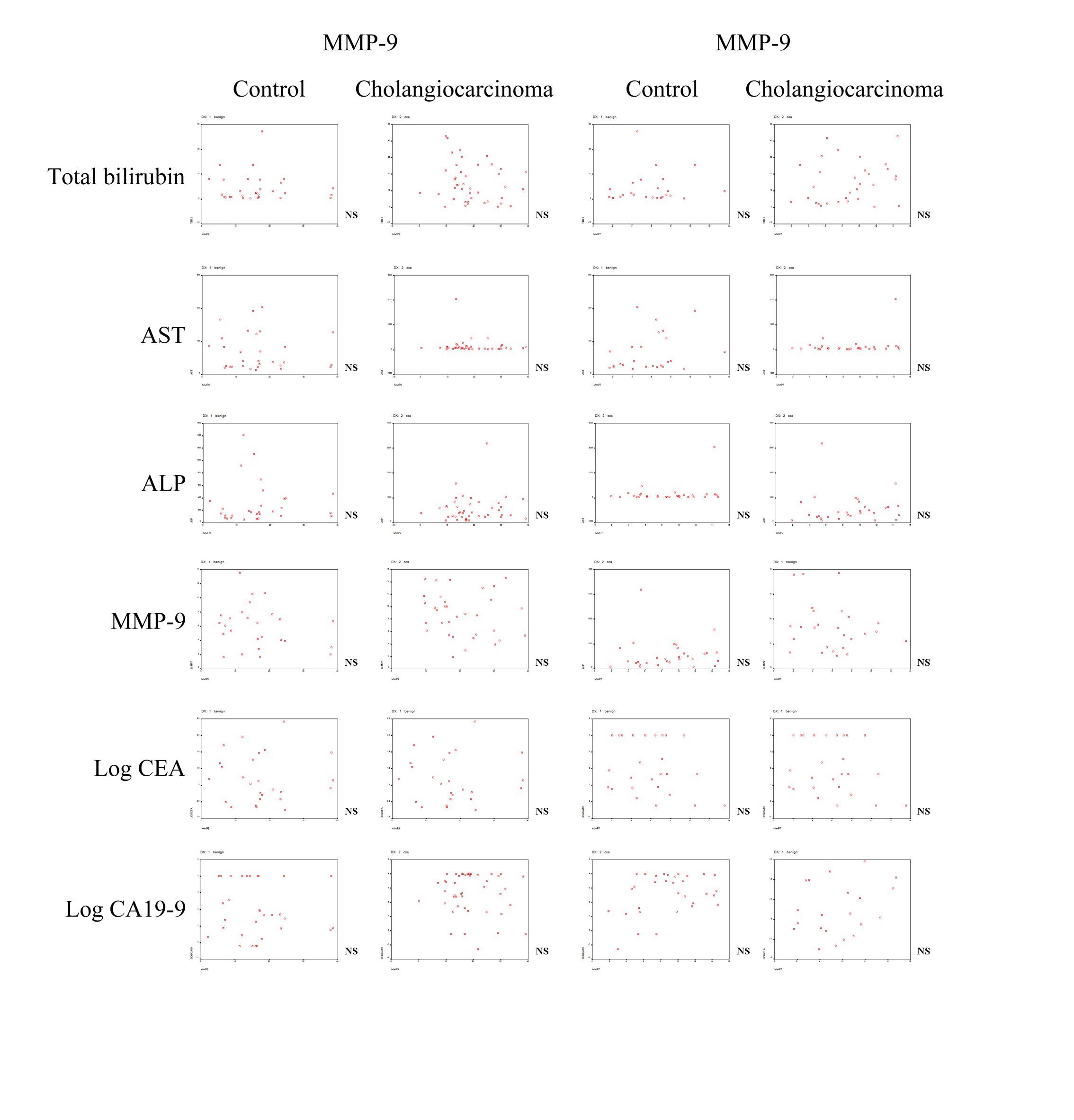

Supplement: Additional file 2 — Scatter plot of the correlation between the blood chemistry values and MMP-9 or MMP-7. A scatter plot was used to identify the correlation between the blood chemistry values (total bilirubin, AST, ALP, Log CEA and Log CA19-9) and MMP-9 or MMP-7 in the control and cholangiocarcinoma patients. This figure demonstrates that there is no significant correlation (p > 0.05) between the blood chemistry values and MMP-9 or MMP-7 in both groups. [file 1471-230X-9-30-S2.tiff]
